# The electronic medical record management systems may improve monitoring and control of disease activity in patients with ankylosing spondylitis

**DOI:** 10.1038/s41598-023-30848-w

**Published:** 2023-03-09

**Authors:** Pei-Ju Huang, Yi-Hsing Chen, Wen-Nan Huang, Yi-Ming Chen, Kuo-Lung Lai, Tsu-Yi Hsieh, Wei-Ting Hung, Ching-Tsai Lin, Chih-Wei Tseng, Kuo-Tung Tang, Yin-Yi Chou, Yi-Da Wu, Chin-Yin Huang, Chia-Wei Hsieh, Yen-Ju Chen, Yu-Wan Liao, Yen-Tze Liu, Hsin-Hua Chen

**Affiliations:** 1 Department of Family Medicine, Changhua Christian Hospital, No. 135, Nanxiao Street, Changhua, 500 Taiwan ROC; 2 Division of Allergy, Immunology, and Rheumatology, Department of Internal Medicine, Taichung Veterans General Hospital, No. 1650, Taiwan Boulevard Sect. 4, Taichung, 40705 Taiwan ROC; 3 School of Medicine, National Yang Ming Chiao Tung University, Taipei, Taiwan; 4 Department of Post-Baccalaureate Medicine, College of Medicine, National Chung Hsing University, Taichung, Taiwan; 5 Department of Business Administration, Ling-Tung University, Taichung, Taiwan; 6 Department of Medical Research, Taichung Veterans General Hospital, Taichung, Taiwan; 7 Department of Medical Education, Taichung Veterans General Hospital, Taichung, Taiwan; 8 PhD Program of Business, College of Business, Feng Chia University, Taichung, Taiwan; 9 Department of Industrial Engineering and Enterprise Information, Tunghai University, Taichung, Taiwan; 10 Department of Holistic Wellness, Mingdao University, Changhua, Taiwan; 11 Division of General Medicine, Department of Internal Medicine, Taichung Veterans General Hospital, Taichung, Taiwan; 12 Institute of Biomedical Science and Rong-Hsing Research Center for Translational Medicine, Chung-Hsing University, Taichung, Taiwan; 13 Institute of Public Health and Community Medicine Research Center, National Yang Ming Chiao Tung University, Taipei, Taiwan

**Keywords:** Computational biology and bioinformatics, Immunology, Health care, Rheumatology

## Abstract

To investigate the impact of an electronic medical record management system (EMRMS) on disease activity and the frequency of outpatient visits among patients with ankylosing spondylitis (AS). We identified 652 patients with AS who were followed up for at least 1 year before and after the first Ankylosing Spondylitis Disease Activity Score (ASDAS) assessment and compared the number of outpatient visits and average visit time within 1 year before and after the initial ASDAS assessment. Finally, we analyzed 201 patients with AS who had complete data and received ≥ 3 continuous ASDAS assessments at an interval of 3 months, and we compared the results of the second and third ASDAS assessments with those of the first. The number of annual outpatient visits increased after ASDAS assessment (4.0 (4.0, 7.0) vs. 4.0 (4.0, 8.0), *p* < 0.001), particularly among those with a high initial disease activity. The average visit time was reduced within 1 year after ASDAS assessment (6.4 (8.5, 11.2) vs. 6.3 (8.3, 10.8) min, *p* = 0.073), especially among patients whose with an inactive disease activity was < 1.3 (ASDAS C-reactive protein (CRP) 6.7 (8.8, 11.1) vs. 6.1 (8.0, 10.3) min, *p* = 0.033; ASDAS erythrocyte sedimentation rate (ESR) 6.4 (8.7, 11.1) vs. 6.1 (8.1, 10.0) min, *p* = 0.027). Among patients who received at least three ASDAS assessments, the third ASDAS-CRP tended to be lower than the first (1.5 (0.9, 2.1) vs. 1.4 (0.8, 1.9), *p* = 0.058). The use of an EMRMS increased the frequency of ambulatory visits among AS patients with high and very high disease activity and reduced the visit time among those with an inactive disease. Continual ASDAS assessments may help control the disease activity of patients with AS.

## Introduction

Ankylosing spondylitis (AS) is a form of axial spondyloarthritis (axSpA), characterized by chronic back pain with articular and periarticular extraspinal features, including synovitis, enthesitis and dactylitis, and nonarticular features, including psoriasis, uveitis, and inflammatory bowel disease (IBD). AS is characterized by sacroiliitis and spinal abnormalities, strongly associated with human leukocyte antigen (HLA) B27 and often accompanied by elevated C-reactive protein (CRP)^1^.

The mean AS prevalence per 10,000 population (from 36 eligible studies) was 0.238% in Europe, 0.167% in Asia, 0.319% in North America, 0.102% in Latin America, and 0.074% in Africa in 2014 systemic research^2^. The prevalence in Taiwan is 0.337%^3^. The risk of AS is higher in men and in individuals with a family history of AS. The guidelines of the European League Against Rheumatism recommend a range of treatment strategies for the optimal management of axSpA, including nonpharmacological treatment, pharmacological treatment, surgery, and lifestyle modifications^4^. The primary goal of AS treatment is to attenuate inflammation for relieving pain and stiffness, preventing or delaying complications and spinal deformity, reducing extraspinal and extra-articular manifestations and comorbidities, and maintaining effective psychosocial function. These AS treatment strategies, along with regular monitoring of disease activity, are generally applied by rheumatologists. The Ankylosing Spondylitis Disease Activity Score (ASDAS) is used as a measure of disease activity in patients with AS by using clinical laboratory data and a self-administered questionnaire. The management of axSpA in Taiwan is strongly influenced by the National Health Insurance reimbursement system and local health circumstances^5^. However, rheumatologists in Taiwan are usually operating at maximum capacity, and consequently, they are unable to assess disease activity in patients with AS. Therefore, a new integrated disease surveillance strategy must be developed to monitor disease activity in patients with AS.

With advances in mobile technologies, electric health (eHealth) and smartphone applications (known as apps) have been developed to facilitate the transmission of information related to infectious diseases in numerous low-income countries, such as some nations in Africa^6^. Furthermore, smart apps have been extensively used for standard clinical evaluations and monitoring diseases and changes in the health status of patients with chronic health conditions^7^, including asthma^8^, obesity, diabetes^9^, hypertension, cardiovascular disease^10^, and multiple sclerosis^11^. Such app-based data systems ensure complete and timely data collection^12^. However, a comprehensive, high-quality, evidence-based data app for disease management in patients with AS is lacking^13^.

Therefore, the purpose of this study was to investigate the effects of an interactive electronic medical record management system (EMRMS) intervention on the disease activity and frequency of outpatient visits of patients with AS in Taiwan.

## Methods

### Ethics

The study protocol was approved by the Institutional Review Board (IRB) of Taichung Veterans General Hospital (TCVGH-IRB No.: CE20145B). All experiments were performed in accordance with relevant guildlines and regulations. Informed consent was waived by Institutional Review Board (II), TCVGH, because individual information had been anonymized, de-identify and de-link before analysis.

### Study design

This was a single center, retrospective, cross-sectional study.

### Data source

The EMRMS was established in November, 2016 to assist rheumatologists in conducting ASDAS assessments and comprehensively evaluating clinical outcomes in all patients with AS attending TCVGH. The EMRMS database contains information necessary to determite ASDAS, including CRP, level and erythrocyte sedimentation rate [ESR], patient comorbidities, patient history, and family history. The reliability and validity of the data have been verified^14^.Patients with AS were consecutively enrolled in the TCVGH-AS cohort after they received a confirmed AS diagnosis from a TCVGH rheumatologist according to the 1984 modified New York criteria^10^. The CRP and ESR data were automatically uploaded to the TCVGH healthcare information system (HIS) to reduce human error. The baseline information, which was collected by trained nurses during the initial visit, including clinical characteristics, onset age, comorbidities at presentation (hypertension, diabetes mellitus, hyperlipidemia, hepatitis B, hepatitis C, renal insufficiency, gout, coronary artery disease, stroke, periodontal disease, osteoporosis, and tuberculosis history), periarticular extraspinal features (synovitis, enthesitis, and dactylitis) and nonarticular manifestations (psoriasis, uveitis, and IBD), family history of autoimmune disease, and patient history of arthropathy, obtained through standardized questionnaires and worksheets to ensure reproducibility and adherence to good laboratory practice. The rheumatologist in charge then confirmed patients’ clinical characteristics, and nurses assisted the patients with AS to complete the self-assessment questionnaires for disease evaluation. The following measures were used: global assessment of disease activity on a numerical rating scale (NRS) of 0–10, back pain on an NRS of 0–10, duration of morning stiffness on an NRS of 0–10, and peripheral pain or swelling on an NRS of 0–10. Before every 3-month visiting clinic, the patient would first to have blood examination. Blood reports can be uploaded to EMRMS through the HIS system, trained nurses assist patient fills out the questionnaire on EMRMS, the assessment of disease activity completed before visiting the doctor. All laboratory data, including CRP and ESR, have been uploaded to the HIS. The IT at TCVGH help "feed-forward" the patient reported outcomes to HIS, and do the auto-calculation of ASDAS-ESR, ASDAS-CRP using the ESR, CRP data in HIS, then "feed-back" these data to both HIS and EMRMS, showing the data on the summary overview "dashboard" in the EMRMS, which was shown both in HIS and the devices (iPAD handled by a nurse in charge and smartphones of patients with AS).

### Definition of AS

Patients were defined as having AS if they received a diagnosis of AS (*International Classification of Diseases, Ninth Revision, Clinical Modification* [*ICD-9-CM*] code 720.0) according to the modified New York criteria for AS proposed in 1984 during at least three ambulatory visits and received AS treatment concurrently.

### Definition of AS disease activity

Four disease activity states for the ASDAS score have been defined: The ASDAS assessment measures four disease activity states: inactive, moderate, high, and very high. Disease status was evaluated on the basis of three cutoff values: 1.3, 2.1, and 3.5 units. The 3 cut-offs selected to separate these states were: < 1.3 between "inactive disease" and "moderate disease activity", < 2.1 between "moderate disease activity" and "high disease activity", and > 3.5 between "high disease activity" and "very high disease activity". The following cutoff values were selected to indicate improvement: a change of ≥ 1.1 units denoted a clinically important improvement, and a change of ≥ 2.0 units denoted a major improvement^15–18^.

### Study participants

A total of 652 eligible patients with AS with complete baseline demographic and assessment data who received an AS diagnosis before April 17, 2020, were enrolled to investigate the changes in their health-related behaviors after EMRMS implementation. Patients with AS with (1) incomplete details related to ASDAS-CRP, ASDAS-ESR, and age of symptom onset in the assessment questionnaires, (2) their first ASDAS assessment after February 01, 2019, (3) or no outpatient visits within 1 year before or after the first assessment were excluded from this study. Of the 652 patients with AS, 201 underwent three consecutive assessments of disease activity using the EMRMS.

### Outcome

The primary outcomes were the frequency and time of outpatient visits. The secondary outcome was changes in ASDAS after the EMRMS intervention.

### Statistical analysis

Continuous variables are reported as means ± standard deviations, and categorical variables are reported as percentages. Differences in continuous variables were assessed for the same patient at two time points by using a paired *t* test. Shapiro–Wilk/Kolmogorov–Smirnov test was used for normality test, non-parametric data was used Wilcoxon signed rank test to compare paired data*.* The results of a cross-validation analysis strongly supported the selected cutoff values^15–18^. Data were analyzed using SAS software (SAS Institute, Inc., Cary, NC, USA).

## Results

**Figure 1:**
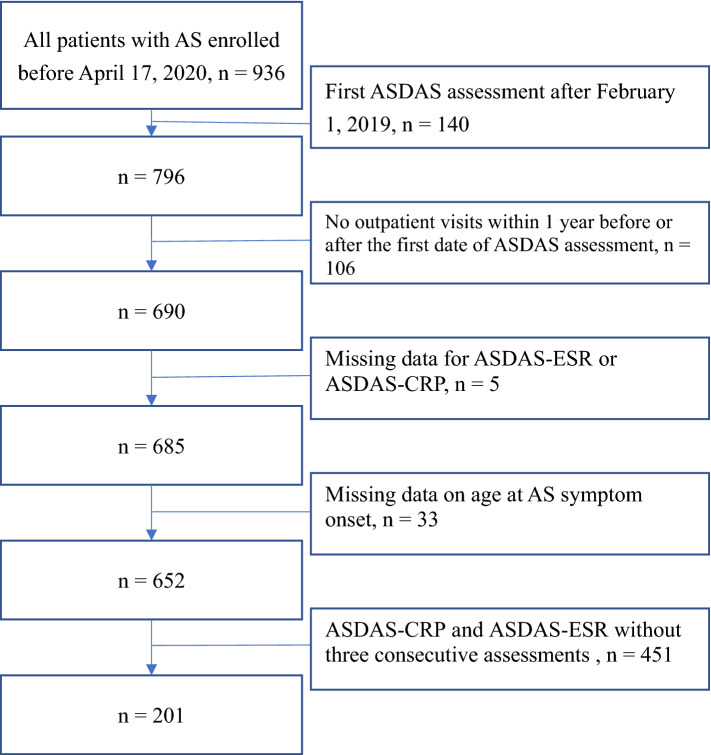
Flowchart of patient enrollment.

We enrolled 652 patients with AS who were followed up for at least 1 year before and after their first ASDAS assessment (Fig. 1) and compared the number of outpatient visits and average visit time within one year before and after the initial ASDAS assessment. We identified 201 AS patients who received ≥ 3 continuous ASDAS assessment with at an interval of 3 months and compared the results of the second and third ASDAS assessments with those of the first.

### Basline characterisitics

The mean age of the 652 eligible patients with AS at their first assessment on the EMRMS was 43.1 ± 13.7 years, and 475 patients were men (72.9%). The AS onset age distribution was 26.8 ± 11.5 years, the disease duration was 16.4 ± 11.7 years, 430 patients were HLA-B27 positive, and 203 were undergoing biologic treatment (31.1%). The most common comorbidity was hypertension (131 patients), the most common AS symptom was uveitis (168 patients), and the most common cause of past history was fracture (70 patients, 10.7%). The mean age of the 201 eligible patients with AS and more than three consecutive assessments on the EREMS was 43.3 ± 13.4 years. In all, 148 were men (73.6%), the AS onset age distribution was 27.0 ± 11.2 years, the disease duration was 16.3 ± 11.6 years, 136 were HLA-B27 positive, and 49 were undergoing biologic treatment (24.4%). The most common comorbidity was hypertension (43 patients), the most common AS symptom was uveitis (53 patients), and the most common cause of past history was fracture (15 patients) Table 1.

**Table 1:** Baseline characteristics of patients with AS obtained using the electronic medical record management system for ASDAS assessment.

	AS patients, n = 652	Eligible AS patients with ≥ 3 continuous assessment, n = 201
Age at first ASDAS assessment, years, Mean ± SD (n/N)	43.1 ± 13.7 (652/652)	43.3 ± 13.4 (201/201)
Gender		
Female	27.1 (177/652)	26.4 (53/201)
Male	72.9 (475/652)	73.6 (148/201)
Age at AS diagnosis, years, mean ± SD (n/N)	26.8 ± 11.5 (652/652)	27.0 ± 11.2 (200/201)
Disease duration, years, mean ± SD (n/N)	16.4 ± 11.7 (652/652)	16.3 ± 11.6 (200/201)
HLA-B27 positive*	88.7 (430/485)	86.1 (136/158)
Biologics therapy	31.1 (203/652)	24.4 (49/201)
Comorbidities*		
Hypertension, % (n/N)	20.2 (131/648)	21.5 (43/200)
Diabetes mellitus, % (n/N)	7.4 (48/650)	4.5 (9/200)
Hyperlipidemia, % (n/N)	14.9 (96/644)	16.2 (32/197)
Hepatitis B, % (n/N)	10.9 (71/649)	9.5 (19/200)
Hepatitis C, % (n/N)	2.3 (15/650)	3.0 (6/200)
Renal insufficiency, % (n/N)	3.2 (21/651)	2.0 (4/200)
Gout, % (n/N)	4.5 (29/651)	3.0 (6/200)
Coronary artery disease, % (n/N)	3.2 (21/650)	3.5 (7/200)
Stroke, % (n/N)	0.3 (2/651)	0.5 (1/200)
Periodontitis, % (n/N)	18.7 (121/646)	19.6 (39/199)
Osteoporosis, % (n/N)	6.7 (42/630)	6.2 (12/193)
Tuberculosis history, % (n/N)	6.9 (45/648)	6.1 (12/198)
AS-related manifestations		
Uveitis, % (n/N)	25.8 (168/651)	26.8 (53/198)
Psoriasis, % (n/N)	6.8 (44/649)	11.1 (22/199)
Crohn's disease, % (n/N)	0.0 (0/650)	0.5 (1/200)
Ulcerative colitis, % (n/N)	0.5 (3/651)	1.0 (2/199)
Peripheral arthritis, % (n/N)	19.3 (125/646)	21.5 (43/200)
Enthesitis, % (n/N)	14.4 (92/641)	18.3 (36/197)
Dactylitis, % (n/N)	2.3 (15/652)	2.0 (4/199)
Family history		
AS-First degree relatives, % (n/N)	18.7 (119/636)	15.5 (30/193)
AS-Secondary degree relatives, % (n/N)	29.0 (185/639)	24.5 (48/196)
Psoriasis, % (n/N)	4.2 (27/643)	5.1 (10/196)
Psoriatic arthritis, % (n/N)	0.6 (4/641)	1.0 (2/194)
Uveitis, % (n/N)	4.7 (30/643)	6.1 (12/196)
Crohn’s disease, % (n/N)	0.0 (0/641)	0.0 (0/195)
Ulcerative colitis, % (n/N)	0.3 (2/641)	0.5 (1/195)
Rheumatoid arthritis, % (n/N)	6.0 (38/638)	5.7 (11/194)
Systemic Lupus Erythematosus, % (n/N)	3.0 (19/641)	2.6 (5/195)
Sicca syndrome, % (n/N)	2.5 (16/641)	3.1 (6/195)
Past history		
Total hip replacement, % (n/N)	3.8 (25/652)	2.0 (4/200)
Total knee replacement, % (n/N)	0.6 (4/652)	0.5 (1/200)
Fracture, % (n/N)	10.7 (70/652)	7.5 (15/200)
Palindromic rheumatism, % (n/N)	1.1 (7/652)	0.5 (1/200)

Abbreviations: *AS* ankylosing spondylitis, *ASDAS* Ankylosing Spondylitis Disease Activity Score.

### Primary outcomes

After the first assessment on the EREMS within 1 year, the frequency of outpatient visits increased from 4.0 (4.0, 7.0) to 4.0 (4.0, 8.0) (*p* < 0.001), particularly in patients with a high disease activity (ASDAS-CRP, 4.0 (4.0, 7.0) vs 4.0 (4.0, 8.0), *p* = 0.001 and ASDAS-ESR, 3.0 (4.0, 7.0) vs 4.0 (4.0, 9.0), *p* < 0.001) and very high disease activity (ASDAS-CRP, 3.0 (5.5, 8.0) vs 5.0 (9.0, 12.0), *p* < 0.001 and ASDAS-ESR, 3.0 (4.0, 8.0) vs 5.0 (8.0, 12.0), *p* = 0.002; Table 2). The duration of outpatient visits decreased from 6.4 (8.5, 11.2) to 6.3 (8.3, 10.8), (*p* = 0.073), especially in those with an inactive disease (ASDAS-CRP, 6.7 (8.8, 11.1) vs 6.1 (8.0, 10.3) min, *p* = 0.033; ASDAS-ESR, 6.4 (8.7, 11.1) vs 6.1 (8.1, 10.0) min, *p* = 0.027; Table 3).

**Table 2:** Frequency of outpatient visits before and after the first EMRMS assessment (n = 652).

			(Min, Max)		(mean ± SD)		Median (P25, P75)		*p*-value of normality test*	*p*-value^#^
Analysis population	ASDAS	Number	One year before assessment	One year after assessment	One year before assessment	One year after assessment	One year before assessment	One year after assessment		
CRP	< 1.3	211	(1.0, 13.0)	(1.0, 15.0)	5.5 ± 3.4	5.6 ± 3.4	4.0 (4.0, 7.0)	4.0 (4.0, 7.0)	< 0.010	0.388
	1.3—< 2.1	245	(1.0, 15.0)	(1.0, 14.0)	5.4 ± 3.4	5.6 ± 3.4	3.0 (4.0, 7.0)	4.0 (4.0, 7.0)	< 0.010	0.400
	2.1—3.5	170	(1.0, 15.0)	(1.0, 18.0)	5.1 ± 3.2	5.9 ± 3.5	4.0 (4.0, 7.0)	4.0 (4.0, 8.0)	< 0.010	0.001
	> 3.5	26	(1.0, 12.0)	(1.0, 15.0)	5.7 ± 3.4	8.7 ± 4.0	3.0 (5.5, 8.0)	5.0 (9.0, 12.0)	0.001	< 0.001
ESR	< 1.3	195	(1.0, 14.0)	(1.0, 13.0)	5.4 ± 3.2	5.4 ± 3.1	4.0 (4.0, 6.0)	4.0 (4.0, 7.0)	< 0.010	0.858
	1.3—< 2.1	258	(1.0, 13.0)	(1.0, 18.0)	5.3 ± 3.4	5.5 ± 3.4	3.0 (4.0, 7.0)	4.0 (4.0, 7.0)	< 0.010	0.151
	2.1—3.5	174	(1.0, 15.0)	(1.0, 15.0)	5.4 ± 3.5	6.2 ± 3.7	3.0 (4.0, 7.0)	4.0 (4.0, 9.0)	< 0.010	< 0.001
	> 3.5	25	(1.0, 12.0)	(1.0, 15.0)	5.6 ± 3.6	8.4 ± 3.9	3.0 (4.0, 8.0)	5.0 (8.0, 12.0)	0.073	0.002
Total		652	(1.0, 15.0)	(1.0, 18.0)	5.4 ± 3.4	5.8 ± 3.5	4.0 (4.0, 7.0)	4.0 (4.0, 8.0)	< 0.010	< 0.001

*Normality test: When number is ≤ 50, the Shapiro–Wilk test is used; when number is > 50, the Kolmogorov–Smirnov test is used.

^#^When normality test *p* value is > 0.05, the pair*-t* test is used; when normality test *p* value is < 0.05, the Wilcoxon Signed Rank test is used.

Abbreviations: *ASDAS* Ankylosing Spondylitis Disease Activity Score, *CRP* C-reactive protein, *ESR erythrocyte sedimentation rate.*

**Table 3:** Time of outpatient visits before and after the first EMRMS assessment (n = 652).

			Time of outpatient visits (minutes)							
			(Min, Max)		Mean ± SD		Median (P25, P75)		*p*-value of normality test*	*p*-value^#^
Analysis population	ASDAS	Number	One year before assessment	One year after assessment	One year before assessment	One year after assessment	One year before assessment	One year after assessment		
CRP	< 1.3	211	(1.5, 41.8)	(0.3, 22.5)	9.2 ± 4.2	8.5 ± 3.3	6.7 (8.8, 11.1)	6.1 (8.0, 10.3)	< 0.010	0.033
	1.3—< 2.1	245	(0.8, 31.6)	(1.0, 37.0)	9.3 ± 4.8	9.0 ± 4.4	6.3 (8.5, 11.2)	6.4 (8.5, 11.0)	< 0.010	0.395
	2.1—3.5	170	(0.9, 22.0)	(0.9, 21.5)	8.7 ± 4.1	8.5 ± 3.4	6.0 (8.3, 11.3)	6.3 (8.1, 10.5)	< 0.010	0.825
	> 3.5	26	(1.2, 17.3)	(4.3, 15.0)	9.6 ± 4.1	9.8 ± 2.7	6.7 (10.0, 11.6)	8.8 (9.7, 11.5)	0.284	0.827
ESR	< 1.3	195	(1.4, 41.8)	(1.7, 22.5)	9.1 ± 4.2	8.4 ± 3.3	6.4 (8.7, 11.1)	6.1 (8.1, 10.0)	< 0.010	0.027
	1.3—< 2.1	258	(0.8, 31.6)	(0.3, 37.0)	8.9 ± 4.2	8.5 ± 4.1	6.3 (8.4, 10.8)	6.0 (7.7, 10.7)	0.015	0.318
	2.1—3.5	174	(1.1, 29.5)	(0.9, 24.1)	9.3 ± 4.6	9.4 ± 3.7	6.2 (8.4, 11.5)	6.8 (9.0, 11.5)	< 0.010	0.623
	> 3.5	25	(3.3, 22.0)	(3.2, 15.0)	11.0 ± 4.5	9.3 ± 2.9	7.7 (11.0, 13.3)	8.2 (9.3, 11.5)	0.610	0.124
Total		652	(0.8, 41.8)	(0.3, 37.0)	9.2 ± 4.4	8.7 ± 3.8	6.4 (8.5, 11.2)	6.3 (8.3, 10.8)	< 0.010	0.073

*Normality test: When number is ≤ 50, the Shapiro–Wilk test is used; when number is > 50, the Kolmogorov–Smirnov test is used.

^#^When normality test *p* value is > 0.05, the pair*-t* test is used; when normality test *p* value is < 0.05, the Wilcoxon Signed Rank test is used.

Abbreviations: *ASDAS* Ankylosing Spondylitis Disease Activity Score, *CRP* C-reactive protein, *ESR* Erythrocyte sedimentation rate.

### Secondary outcomes

The third ASDAS-CRP and ASDAS-ESR tend to be lower than the first assessment (1.5 (0.9, 2.1), 1.4 (0.8, 1.9), *p* = 0.058 and 1.4 (1.1, 2.0), 1.4 (1.0, 1.9), *p* = 0.161, respectively), but both ASDAS-CRP and ASDAS-ESR did not reached significant difference (Table 4).

**Table 4:** ASDAS-CRP and ASDAS-ESR from three consecutive assessments^†^ (n = 201).

	T1	T2	T3	*p*-value^#^		
				T1 versus T2	T2 versus T3	T1 versus T3
ASDAS-CRP						
(Min, Max)	(0.1, 4.1)	(0.0, 4.7)	(0.1, 4.3)	0.225	0.580	0.058
Mean ± SD	1.6 ± 0.8	1.5 ± 0.8	1.5 ± 0.8			
Median (P25, P75)	1.5 (0.9, 2.1)	1.3 (0.9, 1.9)	1.4 (0.8, 1.9)			
ASDAS-ESR						
(Min, Max)	(0.3, 4.4)	(0.3, 4.2)	(0.4, 4.2)	0.510	0.488	0.161
Mean ± SD	1.6 ± 0.8	1.5 ± 0.7	1.5 ± 0.7			
Median (P25, P75)	1.4 (1.1, 2.0)	1.5 (1.0, 2.0)	1.4 (1.0, 1.9)			

Abbreviations: *ASDAS* Ankylosing Spondylitis Disease Activity Score, *CRP* C-reactive protein, *ESR* erythrocyte sedimentation rate, *T* time, *SD* standard deviation, *P* percentile.

^†^Comorbidities were identified within 1 year before the index date.

^#^Because the *p*-value of the normality test (Kolmogorov–Smirnov test) is < 0.05, the Wilcoxon Signed Rank test is used.

^†^Time of the three consecutive assessment: T1: the earliest time was considered the first time; T2: the second time was 84 ± 7 days; T3: the third time was 168 ± 7 days.

## Discussion

To our knowledge, several meta-analyses have assessed the effectiveness of mHealth applications for monitoring AS disease activity in multiple centers^5,6^. However, no study had explored the influence of EMRMS intervention on disease activity and the time and frequency of outpatient visits among patients with AS in a single medical center. This study demonstrated changes in the health-related behaviors of patients with AS, including increased outpatient visit frequency among AS patients with high and very high disease activity, as well asdecreased outpatient visit time among AS patients with inactive disease activity.

The study findings indicate that the proposed smartphone-based management system is a time- and cost-effective disease management tool, achieving high ASDAS and the efficient detection of inflammatory markers in a Chinese AS cohort^17,18^. Self-report ASDAS questionnaires have been applied extensively to evaluate the disease activity of patients with AS in single medical center studies^20–25^. In contrast to previous studies, the current study linked the TCVGH HIS with smartphone applications, wherein laboratory data are automatically integrated into the app and trained nurses assist patients with completing assessments through an NRS on the app for ASDAS calculation. Nobody calls AS patients for additional visit. All treatment decisions, including frequency of follow-up, were left to the physician's discretion. Visits to assess study ASDAS were scheduled at least every 12 weeks. Given that the TCVGH is a center of excellence endorsed by the Asia Pacific League of Associations for Rheumatology, we assume that most rheumatologists at the TCVGH tried to apply the recommendations of treat to target published in 2017 on line first after monitoring of ASDAS in their usual care^26^. In addition, the results of the TICOSPA trial that aimed to assess the efficacy of a tight-control and treat-to-target strategy in axial spondyloarthritis showed that patients with 'usual care' also showed a significant improvement of disease activity^27^.

This system has a high interrater reliability, accuracy, and precision. Optimal treatment of AS must involve shared decision-making between patients and health professionals. Through this user-friendly EMRMS, patients with AS can more thoroughly understand their disease severity, which may in turn improve their treatment adherence rates and increase clinic visits. This study comprehensively furnished many disease information, such as comorbidities, medications, laboratory data, family history and past history. The study findings provide new insight into the use of apps for disease monitoring to reduce consultation times for individuals with a mild disease status, improve the treatment adherence rates of individuals with a severe disease status, and ameliorate AS disease activity.

Our study has some limitations. First, data were collected from a single medical center in Taiwan, which may have introduced selection bias. Second, we did not have the data of changes in disease activity and frequency of outpatient visits in AS patients who did not receive ASDAS assessment using the EMRMS. Therefore, we cannot compare our data of the VGHTC-AS cohort with those of a control group. Third, We need longer follow-up period to investigate the alteration of ASDAS-CRP status in patients who received continual regular assessment using the EMRMS. Finally, the results may not be generalizable to the entire population of Taiwan with AS.

## Conclusions

This is the first single medical center study in Taiwan to compare the treatment outcomes of patients with AS after EMRMS management. The EMRMS is an effective management system that offers satisfactory levels of usability; the data obtained are of a high quality, and the system enables a comprehensive analysis of patient function, helping patients more accurately understand their conditions and thus leading to improved patient treatment adherence. The EMRMS help increase the frequency of outpatient visits in those with high disease activity and may improve control of disease activity in patients with AS. Further research should explore the application of EMRMS for the management of other chronic diseases.

## Data Availability

The datasets generated during the current study are available from the corresponding author on reasonable request.

## References

[1] Sepriano A, Ramiro S, van der Heijde D, van Gaalen F, Hoonhout P, Molto A, Saraux A, Ramonda R, Dougados M, Landewé R (2020). What is axial spondyloarthritis? A latent class and transition analysis in the SPACE and DESIR cohorts. Ann. Rheum. Dis..

[2] Dean LE, Jones GT, MacDonald AG, Downham C, Sturrock RD, Macfarlane GJ (2014). Global prevalence of ankylosing spondylitis. Rheumatol. (Oxf.).

[3] Chou CT, Pei L, Chang DM, Lee CF, Schumacher HR, Liang MH (1994). Prevalence of rheumatic diseases in Taiwan: A population study of urban, suburban, rural differences. J. Rheumatol..

[4] Ramiro S, Nikiphorou E, Sepriano A, Ortolan A, Webers C, Baraliakos X, Landewé RBM, Van den Bosch FE, Boteva B, Bremander A, Carron P, Ciurea A, van Gaalen FA, Géher P, Gensler L, Hermann J, de Hooge M, Husakova M, Kiltz U, López-Medina C, van der Heijde D (2023). ASAS-EULAR recommendations for the management of axial spondyloarthritis: 2022 update. Ann. Rheum. Dis..

[5] Wei JC, Liu CH, Tseng JC, Hsieh LF, Chen CH, Chen HH, Chen HA, Chen YC, Chou CT, Liao HT, Lin YC, Luo SF, Yang DH, Yeo KJ, Tsai WC, Taiwan Rheumatology Association (TRA) (2020). Taiwan Rheumatology Association consensus recommendations for the management of axial spondyloarthritis. Int. J. Rheum. Dis..

[6] El-Khatib Z, Shah M, Zallappa SN, Nabeth P, Guerra J, Manengu CT, Yao M, Philibert A, Massina L, Staiger CP, Mbailao R, Kouli JP, Mboma H, Duc G, Inagbe D, Barry AB, Dumont T, Cavailler P, Quere M, Willett B, Reeder B (2018). SMS-based smartphone application for disease surveillance has doubled completeness and timeliness in a limited-resource setting - evaluation of a 15-week pilot program in Central African Republic (CAR). Confl. Health.

[7] Moses JC, Adibi S, Shariful Islam SM, Wickramasinghe N, Nguyen L (2021). Application of smartphone technologies in disease monitoring: A systematic review. Healthc. (Basel, Switz.).

[8] Marcano Belisario JS, Huckvale K, Greenfield G, Car J, Gunn LH (2013). Smartphone and tablet self management apps for asthma. Cochrane Datab. Syst. Rev..

[9] Wang Y, Xue H, Huang Y, Huang L, Zhang D (2017). A systematic review of application and effectiveness of mHealth interventions for obesity and diabetes treatment and self-management. Adv. Nutrit. (Bethesda, Md.).

[10] Coorey GM, Neubeck L, Mulley J, Redfern J (2018). Effectiveness, acceptability and usefulness of mobile applications for cardiovascular disease self-management: Systematic review with meta-synthesis of quantitative and qualitative data. Eur. J. Prev. Cardiol..

[11] Bonnechère B, Rintala A, Spooren A, Lamers I, Feys P (2021). Is mHealth a useful tool for self-assessment and rehabilitation of people with multiple sclerosis? A systematic review. Brain Sci..

[12] Debon R, Coleone JD, Bellei EA, De Marchi A (2019). Mobile health applications for chronic diseases: A systematic review of features for lifestyle improvement. Diabetes Metab. Syndr..

[13] Ji X, Wang Y, Ma Y, Hu Z, Man S, Zhang Y, Li K, Yang J, Zhu J, Zhang J, Huang F (2019). Improvement of disease management and cost effectiveness in Chinese patients with ankylosing spondylitis using a smart-phone management system: A prospective cohort study. Biomed. Res. Int..

[14] Chen HH, Chen YM, Lai KL, Hsieh TY, Hung WT, Lin CT, Tseng CW, Tang KT, Chou YY, Wu YD, Huang CY, Hsieh CW, Huang WN, Chen YH (2020). Gender difference in ASAS HI among patients with ankylosing spondylitis. PLoS ONE.

[15] van der Linden S, Valkenburg HA, Cats A (1984). Evaluation of diagnostic criteria for ankylosing spondylitis. A proposal for modification of the New York criteria. Arthr. Rheum..

[16] van der Heijde D, Lie E, Kvien TK, Sieper J, Van den Bosch F, Listing J, Braun J, Landewé R, Assessment of SpondyloArthritis international Society (ASAS) (2009). ASDAS, a highly discriminatory ASAS-endorsed disease activity score in patients with ankylosing spondylitis. Ann. Rheum. Dis..

[17] Machado P, Landewé R, Lie E, Kvien TK, Braun J, Baker D, van der Heijde D, Assessment of SpondyloArthritis international Society (2011). Ankylosing Spondylitis Disease Activity Score (ASDAS): Defining cut-off values for disease activity states and improvement scores. Ann. Rheum. Dis..

[18] Machado PM, Landewé R, Heijde DV, Assessment of SpondyloArthritis international Society (2018). Ankylosing spondylitis disease activity score (ASDAS): 2018 update of the nomenclature for disease activity states. Ann. Rheum. Dis..

[19] Au YL, Wong WS, Mok MY, Chung HY, Chan E, Lau CS (2014). Disease activity assessment in ankylosing spondylitis in a Chinese cohort: BASDAI or ASDAS?. Clin. Rheumatol..

[20] Godfrin-Valnet M, Prati C, Puyraveau M, Toussirot E, Letho-Gyselink H, Wendling D (2013). Evaluation of spondylarthritis activity by patients and physicians: ASDAS, BASDAI, PASS, and flares in 200 patients. Joint Bone Spine.

[21] Wach J, Letroublon MC, Coury F, Tebib JG (2016). Fibromyalgia in spondyloarthritis: effect on disease activity assessment in clinical practice. J. Rheumatol..

[22] López-Medina C, Garrido-Castro JL, Castro-Jiménez J, González-Navas C, Calvo-Gutiérrez J, Castro-Villegas MC, Ortega-Castro R, Escudero-Contreras A, Font-Ugalde P, Collantes-Estévez E (2018). Evaluation of quality of life in patients with axial spondyloarthritis and its association with disease activity, functionality, mobility, and structural damage. Clin. Rheumatol..

[23] Chiowchanwisawakit P, Thaweeratthakul P, Wattanamongkolsil L, Srinonprasert V, Koolvisoot A, Muangchan C, Nilganuwong S, Arromdee E, Katchamart W (2019). Relationship between health-related quality of life and patient acceptable symptom state with disease activity and functional status in patients with ankylosing spondylitis in Thailand. J. Clin. Rheumatol. Pract. Rep. Rheum. Musculoskelet. Dis..

[24] Bedaiwi MK, AlRasheed RF, Bin Zuair A, Alqurtas EM, Baeshen MO, Omair MA (2021). A cross-sectional study on clinical characteristics of Saudi axial spondylarthritis: Preliminary results. Eur. Rev. Med. Pharmacol. Sci..

[25] Rusman T, Nurmohamed MT, Hoekstra S, van Denderen CJ, van Vollenhoven RF, Boers M, Ter Wee MM, van der Horst-Bruinsma IE (2021). Disease activity in women with ankylosing spondylitis remains higher under Tumour Necrosis Factor inhibitor treatment than in men: a five-year observational study. Scand. J. Rheumatol..

[26] Smolen JS, Schöls M, Braun J, Dougados M, FitzGerald O, Gladman DD, Kavanaugh A, Landewé R, Mease P, Sieper J, Stamm T, Wit M, Aletaha D, Baraliakos X, Betteridge N, Bosch FVD, Coates LC, Emery P, Gensler LS, Gossec L, van der Heijde D (2018). Treating axial spondyloarthritis and peripheral spondyloarthritis, especially psoriatic arthritis, to target: 2017 update of recommendations by an international task force. Ann. Rheum. Dis..

[27] Molto A, López-Medina C, Van den Bosch FE, Boonen A, Webers C, Dernis E, van Gaalen FA, Soubrier M, Claudepierre P, Baillet A, Starmans-Kool M, Spoorenberg A, Jacques P, Carron P, Joos R, Lenaerts J, Gossec L, Pouplin S, Ruyssen-Witrand A, Sparsa L, Dougados M (2021). Efficacy of a tight-control and treat-to-target strategy in axial spondyloarthritis: results of the open-label, pragmatic, cluster-randomised TICOSPA trial. Ann. Rheum. Dis..

